# Silicon CMOS architecture for a spin-based quantum computer

**DOI:** 10.1038/s41467-017-01905-6

**Published:** 2017-12-15

**Authors:** M. Veldhorst, H. G. J. Eenink, C. H. Yang, A. S. Dzurak

**Affiliations:** 1 Qutech and Kavli Institute of Nanoscience, TU Delft, 2600 GA Delft The Netherlands; 2 Centre for Quantum Computation and Communication Technology, School of Electrical Engineering and Telecommunications, The University of New South Wales, Sydney, NSW 2052 Australia

## Abstract

Recent advances in quantum error correction codes for fault-tolerant quantum computing and physical realizations of high-fidelity qubits in multiple platforms give promise for the construction of a quantum computer based on millions of interacting qubits. However, the classical-quantum interface remains a nascent field of exploration. Here, we propose an architecture for a silicon-based quantum computer processor based on complementary metal-oxide-semiconductor (CMOS) technology. We show how a transistor-based control circuit together with charge-storage electrodes can be used to operate a dense and scalable two-dimensional qubit system. The qubits are defined by the spin state of a single electron confined in quantum dots, coupled via exchange interactions, controlled using a microwave cavity, and measured via gate-based dispersive readout. We implement a spin qubit surface code, showing the prospects for universal quantum computation. We discuss the challenges and focus areas that need to be addressed, providing a path for large-scale quantum computing.

## Abstract (web summary)

Realisation of large-scale quantum computation requires both error correction capability and a large number of qubits. Here, the authors propose to use a CMOS-compatible architecture featuring a spin qubit surface code and individual qubit control via floating memory gate electrodes.

## Introduction

The most promising routes towards large-scale universal quantum computing all require quantum error correction (QEC)^1^, a technique that enables the simulation of ideal quantum computation using realistic noisy qubits, provided that the errors are below a fault-tolerant threshold. Using the most forgiving methods, such as the two dimensional surface code^2^, these error thresholds can be as high as 1%^3^, a level that is now routinely achieved across several qubit platforms^4–10^. However, these approaches also require a platform that can be scaled up to very large numbers of qubits, of order 10^8^. Developing scalable qubit arrays constitutes one of the most stringent barriers in the field, even for the most promising platforms.

Silicon CMOS integrated circuits (ICs) are the prototypical example for scalable electronic platforms, now holding transistor counts exceeding billions. This remarkable level of integration is based upon decades of advances in silicon materials technologies^11^, and these will also be crucial in the development of high-quality spin qubits. A key architectural aspect of ICs has been the use of parallel addressing via word lines and bit lines facilitating rapid read and write operations on large 2D arrays of bits. Unfortunately, this method cannot directly be applied to scale qubit arrays. Unlike transistors, the tolerance levels of qubits are small, thereby requiring individual tunability.

Here, we show an advanced architecture for parallel addressing of silicon spin qubits and integrating highly repetitive error correction methods like the surface code. In addition, we show that individual qubit stabilization is obtained via floating memory gate electrodes that can be routinely reset, similar to dynamic random access memory (DRAM) systems. Altogether, these allow the design of a platform where the number of addressing lines increases in a scalable manner proportional to $\sqrt N$, where *N* is the number of qubits. While silicon was recognized early on as a promising platform in the seminal work of Kane^12^, leading to many novel architectures^13–19^, a key and contrasting feature of our approach is that each architectural component is based on existing devices and commercially available technology to provide a scalable solution.

## Results

### Physical architecture

**Fig. 1:**
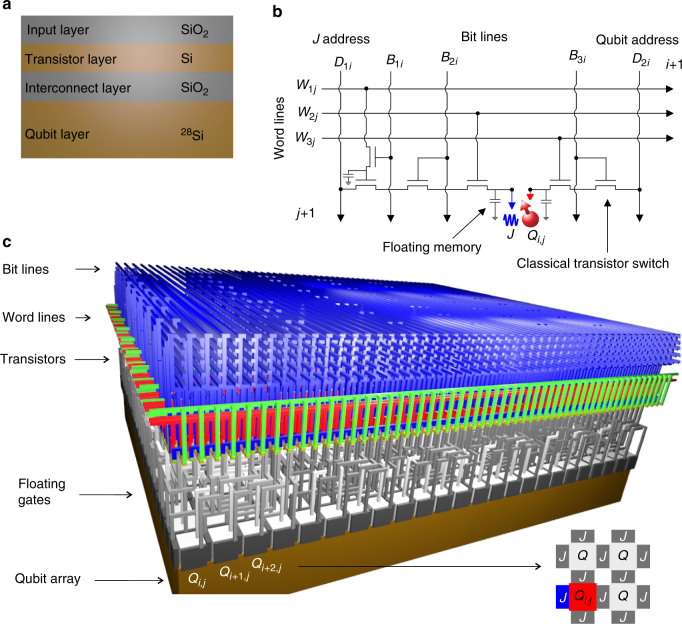
Physical quantum processor. **a** A silicon-on-insulator (SOI) wafer is processed, such that the bottom layer of isotopically enriched silicon-28 contains the 2D qubit array and the top layer of silicon forms the transistors to operate the qubits. These are interconnected through the oxide regions using polysilicon (or other metal) vias. **b** Electrical circuit for the control of one *Q*-gate and one *J*-gate allowing the required individual, row-by-row, or global operations, as explained in the main text. **c** Physical architecture to operate one unit module containing 480 qubits. The inset on the bottom right shows a plan view cross-section through the qubit plane. Each *J* gate and qubit is connected via the circuit shown in **b**

The general architecture we propose is depicted in Fig. 1. We start with a silicon wafer, including an isotopically enriched silicon-28 layer. After CMOS manufacturing, the top layers host the classical circuitry, and the silicon-28 bottom layer holds the quantum circuit. These are interconnected via metal lines which penetrate the oxide region, see Fig. 1a. The fabrication could be performed monolithically, from a single wafer, or include flip-chip technologies to enable the construction of the two circuits separately. We focus here on single spin qubits confined in quantum dots^10^. The tremendous improvements in CMOS technology have resulted in feature sizes that are well below the minimal requirements for quantum dot definition. However, we envision that the small acceptable tolerance levels of qubits will require a certain number of control lines for tunability. In a dense 2D array, this set of requirements will then determine the minimum qubit size for an extendable structure. For complete qubit control, we use a single floating gate for quantum dot definition and a single floating gate for qubit coupling between each qubit. One data line (*D*_2*i*_) is interconnected to each corresponding qubit (*Q*_1_) to tune the qubit resonance frequency (*v*_1_), while a second (*D*_1*i*_) interconnects to each *J*-gate to control the exchange coupling between qubits, shown in Fig. 1b. To provide individual, row, or global qubit addressing, the data lines are controlled by a combination of word lines (*W*) and bit lines (*B*). The required control circuit includes six transistors that connect the data lines via the word lines and bit lines to the floating gates. This circuit is extendable over multiple gates. For simplicity we have shown only one *J*-gate control structure, whereas an extendable structure contains two.

**Fig. 2:**
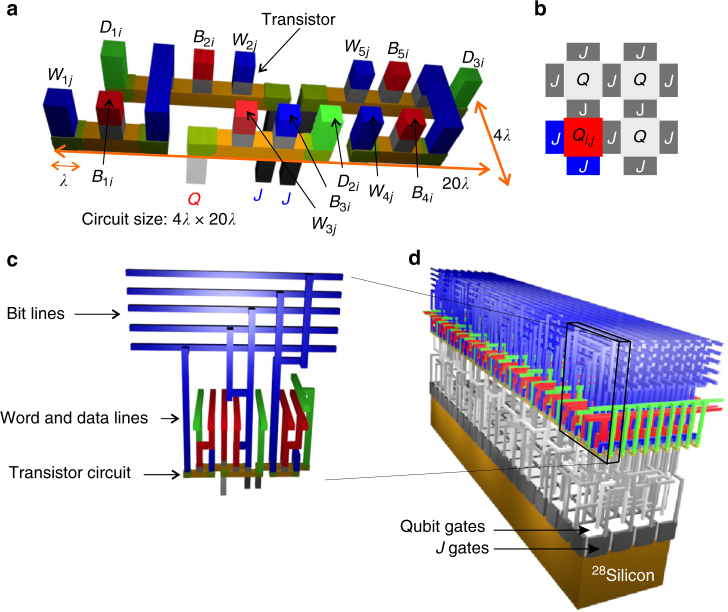
Quantum processor integration scheme. **a** Physical circuit for a single qubit and two *J*-gates (see schematic **b**). The gray elements in **a** correspond to the transistor switches used to activate a line. The scale *λ* is the features size, which is presumed constant for each metal or dielectric layer. **c** Same as **a**, but from a different perspective, in order to show how word, bit, and data lines connect with the unit cell. **d** In order to match the difference in aspect ratios between the qubit layer and control layer, the control elements for a single qubit and two *J*-gates are extended to a 4 × 20 qubit array. Another extension must be made to accommodate for the surface code sequences shown in Fig. 4, so that a single qubit module becomes a 24 × 20 qubit array, as depicted in Fig. 1

The size of a physical circuit for a single extendable element, as shown in Fig. 2a, will be highly dependent on the specific details of the CMOS fabrication process used. However, by assuming the minimal width of, and separation between, the gates and doped regions is equal to the minimum feature size *λ*, the classical circuit occupies an area 80*λ* ^2^ per qubit. A feature size of 7 nm would require a minimum qubit size of ≈63 × 63 nm^2^ (including half the barrier area that separates the qubits), consistent with experimental realizations of silicon quantum dot qubits^10, 20^. Large foundries are now capable of manufacturing some features down to this size, but ongoing advances in down-scaling will be needed to fabricate the classical devices assumed here, and so the development of such a quantum computer will therefore need to proceed hand-in-hand with the ongoing advances in semiconductor technology. For example, the industrial 14 nm node has a transistor fin width of only 8 nm, and a transistor gate pitch of 70 nm (http://www.intel.com/content/dam/www/public/us/en/documents/technology-briefs/bohr-14nm-idf-2014-brief.pdf), consistent with a quantum dot size. Nonetheless, further down-scaling or advances in 3D technology would be needed to place several transistors above a quantum dot^21^. Alternatively, multiple transistors could be stacked in different layers, such that each individual transistor can be larger in size.

Generally, the most compact classical circuits have different geometries from quantum circuits. While a 2D qubit plane takes on a square shape due to square (or circular) shaped qubits, we found that this is generally not the case for the most optimal classical control layers. The situation is further complicated by the geometrical layout of the metal connection lines, determined by the quantum error correction implementation. To overcome the complexity in scaling these differently sized circuit components, we use vertically stacked interconnection layers. After expanding to a large number of qubits, as described below, we can match the aspect ratios of the layers. We start with the basic control structure, which connects to a qubit and two *J*-gates, with the assumed single linewidth parameter *λ*, set by the feature size of the fabrication platform, see Fig. 2. The aspect ratio of the control structure is $4\lambda \times 20\lambda$. In order to match with a square qubit, we extend the control structure to a set of 20 × 9. This control structure addresses a qubit array 20 × 4, which has the same footprint. However, in order to match the surface code protocol discussed in the section Surface code operations, we again have to extend the structure to hold 54 × 9 classical control structures for 24 × 20 qubits (note the presence of 6 redundant classical control structures that are required in order to match the aspect ratio).

As the number of qubits increases, the three layers become spatially identical. This point is reached upon expanding the structure to host 480 qubits, and an entire qubit module is shown in Fig. 1. Beyond this, further scaling becomes a straightforward replication of this 480 qubit module. A full quantum processor would then contain multiple modules and the edges would be connected to a doped silicon region, serving as an electron reservoir, from which electrons may be sequentially loaded into the qubit array as is done in charge-coupled devices^22^. The word and bit lines of the integrated quantum processor chip will then be connected to classical control and measurement electronics^23^ that can reside next to or further away from the quantum chip depending on their level of power dissipation.

### Electrical operation

**Fig. 3:**
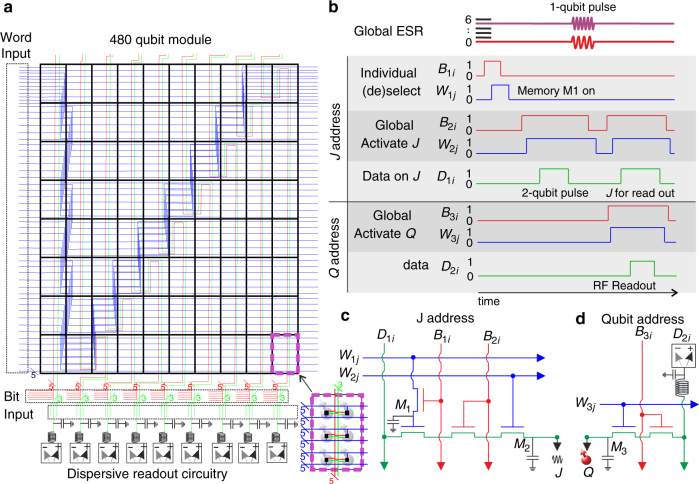
Electrical circuit and qubit addressing scheme. **a** Electrical wiring of the 480 qubit module. The word lines (*W*), bit lines (*B*), and data lines (*D*) can be addressed to enable global control, to couple and readout row-by-row and to individually (de)select qubits. The *W* and *B* lines are grouped in five and the *D* in three, such that a combination of these form the lines of the electrical circuit of a single extendable structure, consisting of a single qubit and two *J* gates. The zigzag structure in **a** is to accommodate for the different aspect ratios of qubit size, control size and in order to be consistent with surface code operation. The purple rectangle displays the region that is occupied by 6 qubits, corresponding to a surface code unit cell (see Fig. 4b). Note that the word lines are connected to the qubits in an alternating arrangement in order to make the circuit compatible with our spin qubit surface code scheme. **b** Typical operation protocol of the electrical circuit shown in **c**, **d**. Individual qubit selection is via lines *W* and *B*_1_ that (de)charge floating electrodes (*M*1 in **c**) and (dis)connect the data lines from the corresponding *J*-gates. Two-qubit operations are performed by activating the associated lines *W*_2_ and *B*_2_ and sending a pulse through data line *D*_1_. Global single-qubit operations can be applied by broadcasting an ESR pulse at the resonance frequencies of the corresponding subgroup of qubits at any time of the sequence. Readout is enabled via the lines *W*_2_, *B*_2_, *W*_2_, and *B*_3_. Then a pulse turns on the selected *J* gates, and RF readout is performed via the data line *D*_2_ connected to the qubit. The electrical circuits in **c**, **d** show the corresponding structures to control the qubits and the exchange coupling between them. The floating memories *M*_1_ and *M*_2_ are to maintain the desired electric fields on the respective *J* and *Q* gates and may be periodically refreshed

**Fig. 4:**
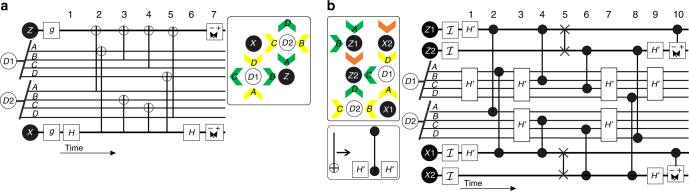
Surface code operation. **a** General surface code operation^3^ and **b** surface code operation for spin qubits. To match this error correction scheme with a spin qubit system, the CNOT gate is decomposed into CPHASE and Hadamard operations, which are elementary operations for quantum dot qubits. CPHASE operations with quantum dot qubits usually result in additional $\hat z$ rotations, which can be corrected using single qubit gates. Here this is included in the Hadamard, resulting in a Hadamard-like operation. Read out is via a parity measurement requiring two quantum dots, such that a unit cell consists of six (schematic in **b**) instead of the usual four (schematic in **a** ^3^) qubits. A single unit cell contains now two data qubits, *D*_1_ and *D*_2_, and four measurement qubits, *Z*_1_, *Z*_2_, *X*_1_, and *X*_2_. Each of these qubit classes has a well-defined independent qubit resonance frequency. To enable all nearest neighbor operations, an additional SWAP operation is included, resulting in protocol **b**. A single cycle of initialization, control, and readout corresponds to 10 steps. Note that the labels *A,B,C*, and *D* refer to the data qubits associated with the respective measurement qubit

We now turn to the electrical operation of the qubit module, Fig. 3, and consider a surface code that is specifically designed for quantum error correction and fault-tolerant operation of this CMOS processor, Fig. 4. We assume that the complete structure is maintained at cryogenic temperatures (∼1 K or less) inside an electron spin resonance (ESR) system, which will be used to apply qubit control pulses. A single electron is loaded into each quantum dot by addressing the corresponding word and bit lines and the electron occupancy is verified by gate-based dispersive readout, as shown in Fig. 3 and described further below. Each qubit must be calibrated to its desired qubit resonance frequency by tuning the associated floating memory gate, using electrical *g*-factor control, as has been demonstrated experimentally^10^. The surface code operation we discuss here requires a total of six different resonance frequencies (see Fig. 4). The need for six qubits instead of the more usual four qubits is because the readout is based on parity, which requires two qubits for measurement, as will be discussed in the section Surface code operations. The qubit gates (*Q*_*ij*_) are calibrated using the data line (*D*_*ij*_) to voltages such that the exchange coupling between adjacent qubits is negligible when the intermediate *J*-gates are set at an “off” bias point, and for which there is a common value of exchange when the *J*-gates are set to an “on” bias. Global (i.e., parallel) control is a crucial aspect for large-scale operation. The use of floating memory gates in the proposed architecture here has the significant advantage of enabling the individual tuning of qubits, while having a minimal number of control lines that can then be set to common bias levels, thus enabling global operations.

### Gate-based dispersive readout and initialization

Two popular methods for spin qubit readout are based on spin to charge conversion: readout based on the Zeeman energy (using a reservoir);^24^ and readout based on the singlet-triplet energy (via Pauli spin blockade)^25^. In tightly confined silicon quantum dots, where the next orbital state is typically several meV above the ground state, the first excited state is the next available valley state, and so the relevant energy for the Pauli spin blockade protocol is largely determined by the valley splitting energy, which can be almost 1 meV^26^. Both approaches can be made compatible with our control circuitry, but readout based on Pauli spin blockade can offer a number of advantages, including: (i) a larger relevant energy scale leading to higher readout fidelity; (ii) no necessity for a large electron reservoir for each qubit; and (iii) a large magnetic field is not required so that the qubit operating frequencies can be much lower, of order one GHz. We therefore propose to use Pauli spin blockade for parity readout between two spin qubits.

Dispersive readout^27–31^ has been considered extensively for multi-dot qubits such as singlet–triplet qubits^25^, but here we envision the readout of single spins by exploiting Pauli spin blockade. Single spin states can be projected onto singlet-triplet states using a reference neighbor dot, thus allowing a parity measurement between two qubits. We prepare the system at large detuning in the singlet (0,2) charge state, where the singlet is the ground state. Consequently, we decrease the detuning and pulse to the (1,1) charge state. Due to the Zeeman energy difference between the two dots, the singlet state evolves into the state where in the dot with the larger *g*-factor the spin state is $\left| \downarrow \right\rangle$ and in the dot with the smaller *g*-factor the spin state is $\left| \uparrow \right\rangle$ and this completes the initialization. In order to avoid transitions to other states, the pulsing speed is limited by the tunnel coupling and Zeeman energy difference between the qubits, which can be larger than 100 MHz^32^.

Qubit readout is based on the reverse process of initialization. We first control the spin of the reference dot (the dot with the larger *g*-factor) to the state $\left| \downarrow \right\rangle$ and then adiabatically pulse to the (0,2) charge state. If the measurement dot is in the state $\left| \downarrow \right\rangle$, the state will remain in the (1,1) charge state due to Pauli spin blockade whereas if the measurement dot is in the state $\left| \uparrow \right\rangle$, the end state will be the singlet with (0,2) charge state. Pulsing close to zero-detuning results in a movement of charge only if the measurement dot is in the state $\left| \uparrow \right\rangle$ and this can be detected using gate-based dispersive readout^27–31^, see Fig. 3. Avoiding spin relaxation will be a particular challenge to achieve high-fidelity, thus requiring a fast protocol and absence of relaxation hot-spots in the pulsing regime^26^.

The readout is performed in a row-by-row manner and the parity analyzers are connected to the data lines *D*_2*i*_ via bias tees, see Fig. 3d. Using classical circuitry, it is possible to frequency multiplex an entire row^33^ so that only one RF analyzer circuit is needed, however the amount of channels will be limited due to crosstalk and finite bandwith. For large qubit numbers, a combination of multiple analyzers, as depicted in Fig. 3a, and temporal multiplexing could provide solutions. Operating dispersive readout at 1 GHz enables readout on timescales of order 10–100 ns, so that a large qubit array could be read out well within the single qubit coherence time of 28 ms in ^28^Si substrates^10^. A combination of these multiplexing schemes can be used depending on available space, frequency bandwidth and time.

To be able to perform parallel operations, an integrated 3D arrangement of the addressing and qubit structures is required, such that a certain combination of word lines and bit lines will address the same particular qubit in each unit cell. This is implemented in the schematic in Fig. 3, with a unit cell of 2 × 3 qubits. This size is based on the required 2 data qubits and 4 measurement qubits for surface code operations using parity readout (explained in section b). For other qubit encoding schemes, different unit cells could be preferable. To deselect individual qubits, the *J*-gates surrounding the relevant qubits are deactivated (see Fig. 3b), thereby isolating them from the data qubits and creating an additional degree of freedom in the array for quantum computation. This protocol will be particularly relevant for operation of the defect-based surface code.

### Surface code operations

Surface codes are among the most promising methods for quantum error correction^1, 3^. The standard surface code cycle and unit cell^3^ are shown in Fig. 4a. The protocol contains a sequence of CNOT operations together with single qubit Hadamards, readout and initialization steps. An alternating arrangement of data and measurement qubits is used, where two data qubits interact with four measurement qubit neighbors. In our approach, we perform readout with spin to charge conversion based on the singlet–triplet energy (via Pauli spin blockade). This parity readout process requires two qubits, and so the surface code unit cell expands to six qubits, as shown in Fig. 4b. This implementation is thus slightly larger than the usual surface code unit cell of four qubits. In order to access all sites, an additional SWAP operation is included (step 5 in Fig. 4b). The CNOT operation is realized by a combination of a CPHASE gate interleaved between two single qubit rotations, shown in Fig. 4b. The CPHASE gate is created by turning the interaction on, such that the qubits will acquire a time-integrated phase dependent on the spin state of the coupled qubit^34^. A SWAP operation can be realized in a similar way, but requires the tunable qubit resonance frequency difference to be much smaller than the interaction strength.

The measurement qubits are initialized to ${\cal I}$ by adiabatically moving from the (0,2) charge state to the (1,1) charge state, as discussed in the section Gate-based dispersive readout and initialization. Single qubit Hadamard operations and the two-qubit CPHASE and SWAP operations are then performed, followed by measurement of the spin states using dispersive readout. This projective measurement of a system of multiple qubits enables non-destructive quantum error correction of single qubits. The complete surface code cycle for quantum dot qubits, see Fig. 4b, then involves ten steps.

The focus of the work presented here is the design of a manufacturable 2D qubit array architecture, and we envision that many different surface code schemes and even analog quantum simulator algorithms can be constructed based on our design. We therefore do not undertake here a detailed analysis of the particular error thresholds associated with our surface code implementation. A new fault-tolerant error threshold will need to be calculated for each particular qubit encoding and manipulation scheme, and this is a crucial challenge that needs to be addressed in future. We expect that the associated fault-tolerant error thresholds can be large, given that the number of operations is comparable with those previously reported^3^. Recent demonstrations of single- and two-qubit gates in silicon^10, 34^ provide significant scope to meet all the required fault-tolerant thresholds. Further improvements in two-qubit fidelities are conceivable, for example via operation at the charge symmetry point for a pair of quantum dot qubits^35, 36^.

To perform logical quantum operations on the qubit module with a defect-based surface code, qubit deselection is required to create holes for braiding operations^3^. Individual qubit (de)selection is enabled by the circuit shown in Fig. 3c, using word and bit lines *W*_1_ and *B*_1*i*_. The required holes will be limited, as most physical qubits will be used to create the logical qubits. The infrequent nature of required qubit (de)selection allows for this to be done individually, rather than globally, and we achieve this by deactivating the associated *J*-gates, thereby isolating the associated data qubits from their measurement qubits.

### Heat dissipation

A critical factor for almost any large-scale computing platform is cooling power. A detailed analysis based on a specific design and targeted operation, going beyond this work, will therefore be highly valuable. Focus areas contributing to the total power dissipation include the dynamic power produced by the *J*-gates. The power dissipation of a single surface code unit cell, shown in Fig. 4b, is $P = CV^2\alpha f$, with *C* the capacitance of the floating memory, *V* the switching voltage, and *α* the activity factor relative to the surface code clock cycle with frequency *f* ≈ 0.1 MHz (assuming Rabi frequencies on the order of 1 MHz^10^). The surface code unit cell is operated using 54 transistors and during a full cycle the *J*-gate actvity *α* = 12. The floating gate electrodes may be periodically refreshed, as in DRAM technology, but we estimate that for high-fidelity qubit operation *RC* times beyond one second will be required to avoid significant drifts during operation. We assume this requires a capacitance *C* ≈1pF, with an associated Johnson–Nyquist thermal noise $V_{{\mathrm{thermal}}} = \sqrt {K_{\mathrm{B}}T/C} \approx$ 1μV, providing a tolerable level^34^. Assuming a switching voltage *V* = 0.2 V results then in a power dissipation for a single unit cell of P≈50 n*W*. This power, however, can be dissipated at a higher temperature stage and superconducting lines can connect the circuit to remote current sources isolating the qubit chip from the dissipation.

Dissipation through leakage, however, can pose a serious challenge and will require significant cooling. Recent experiments using floating gates showed drifts of approximately one Coulomb oscillation per hour (≈8 mV/h)^37^, giving prospects that with frequent refreshing minimal voltage shifts will be caused provided dissipation can be handled. Large dilution refrigerators can already provide more than 1 mW cooling power at 100 mK. The ultimate local cooling power is therefore most likely limited by the thermal conductivity of the circuit. We now consider the cooling from the top through the upper layers of the circuit hosting the addressing lines. The thickness will depend on the exact implementation, but assuming ten to twenty stacked metallic layers we estimate that the total thickness of the lines will be below 5. These lines could be made out of polysilicon with a thermal conductivity *k* = 100 W/m/K at temperatures close to zero Kelvin. The surface code unit cell for spin qubits occupies an area 480 *λ* ^2^, such that for *λ* = 7 nm the available cooling power is ≈500 nW/K per unit cell. Taking the 50 nW estimate of the power dissipation of a unit cell, we thus estimate that the architecture can operate at 100 mK, even if all dynamical power is dissipated at the lowest temperature stage. We note that while this is a rough estimate, silicon metal-oxide-semiconductor (MOS) spin qubits have a significant potential for qubit operation at higher temperatures, due to the large energy scales of their excited states and measured valley splittings, exceeding 10 K^26^. Further reductions in the required cooling power can be made by reducing the operation voltage, which is foreseeable at cryogenic temperatures, but possibly also by utilizing single-electron-transistors for the switching elements^38^, thereby significantly lowering the switching voltage.

A more specific analysis of the dissipated power will need to be done for different layouts, to determine the main contributors and limits. A significant challenge will be the design of nano-sized capacitors; which will likely require a vertical geometry to meet the small feature sizes set by the quantum dot dimensions. Depending on operation temperature, required resolution, and shaped pulses that can reduce sensitivity to noise, capacitor values below 1 could be sufficient. An important engineering challenge will therefore be the optimization and demonstration of capacitors that are comparable in size with the quantum dots.

## Discussion

The conceptual architecture shown here demonstrates that an array of single electron spins confined to quantum dots in isotopically purified silicon can be controlled using a scalable number of control lines. We have shown that the often argued compatibility of silicon spin qubits with standard CMOS technology is non-trivial. However, the proposal presented here for quantum dot qubits, provides scope for fabrication made consistent with standard CMOS technology and opportunities to scale up to thousands or even millions of qubits. Provided that the down-scaling of CMOS transistors continues as anticipated, the control and measurement circuitry described can be integrated with qubits of a size that have already been experimentally demonstrated^10, 20, 34^. The combination of ESR control, exchange coupling and dispersive readout of this design enables surface code operations to be performed using this platform. A key advantage is the possibility of global qubit control, so that many qubits can be addressed within the qubit coherence time.

The proposed architecture is based on the current experimental status of silicon qubits and requires multiple transistors per qubit, significantly challenging CMOS manufacturing capabilities. Advancements in device uniformity and reproducibility could lower the number of required transistors. For example, with more uniform qubits the tuning circuitry and associated floating gates might not be needed. In addition operating at low magnetic fields will result in uniform qubit frequencies, avoiding the need for *g*-factor tuning. This limits functionality, since single-qubit gates can then be applied only globally, but universal computing is still possible using the local two-qubit gates. We anticipate that 2D arrays with such limited functionality can be realized in the near future, and will aid in the development of the universal quantum processor as presented here.

The architectural concept of using floating gates to compensate qubit-to-qubit variations, and the integration of crossbar technology to efficiently address a large qubit array, could be applied to a number of platforms, including spin qubits based on either Si/SiO_2_ or Si/SiGe heterostructures, and adapted for various modes of operation such as single spin qubits^10, 20^, singlet–triplet qubits^39^, exchange-only^40^ or hybrid qubits^41^. The system we considered here requires only local exchange interactions, but the architecture could also be incorporated into larger architectures that include long-range qubit coupling^14, 42–44^, for example, to interconnect quantum structures as presented here. While we consider the fabrication including a single layer of classical elements, a more advanced and complex fabrication process could include multiple stacked layers to allow for more complex classical electronics per qubit, or for a separate control circuit that is purely dedicated for calibration and stability. A more sophisticated design could also include frequency multiplexing along a row, allowing global readout. These are a few of the many opportunities for spin qubits that could provide solutions to the challenges presented here, including the limited available cooling power at lower temperature and the requirement for small feature sizes. While the full fabrication and operation of our architecture is a formidable task, we believe that the identification of the key requirements for a spin qubit quantum computer fully engineered using semiconductor manufacturing paves the way towards an era of large-scale quantum computation; using the same silicon chip technology that has defined our current information age.

### Data availability

The data sets generated during the current study are available from the corresponding authors on reasonable request.
